# The efficacy of Ezetimibe added to ongoing Fibrate-Statin therapy on postprandial lipid profile in the patients with type 2 Diabetes mellitus

**DOI:** 10.1186/2251-6581-12-24

**Published:** 2013-06-04

**Authors:** Faranak Sharifi, Nima Hojeghani, Saeideh Mazloomzadeh, Zahra Shajari

**Affiliations:** 1 Metabolic Diseases Research Centre, Zanjan University of Medical Sciences, Zanjan, Iran; 2 Department of Internal Medicine, Vali asr Hospital, Zanjan, Iran; 3 Zanjan Metabolic Diseases Research Centre, Vali-e-asr Hospital, Zanjan, Iran

**Keywords:** Ezetimibe, Gemfibrozil, Postprandial hypertriglyceridemia, Type 2 diabetes

## Abstract

**Background:**

Postprandial hypertriglyceridemia in diabetes mellitus can be followed by endothelial dysfunction, impaired vascular compliance and increased cardiovascular complications. So focus on better control of postprandial hypertriglyceridemia is as important as controlling fasting triglyceride level in type 2 DM.

**Objective:**

We evaluated the effect of ezetimibe adding to fibrate or statin on postprandial hypertriglyceridemia.

**Methods:**

In a randomized controlled clinical trial, 47 subjects with type 2 diabetes and hypertiglyceridemia were enrolled and divided in three treatment groups including Gemfibrozil 1200_mg/d_ + placebo(group A), Ezetimibe10_mg/d_ + Gemfibrozile 1200_mg/d_(group B) or Ezetimibe10_mg/d_ + Atorvastatin10_mg/d_ (group C) for a 6- week period. Oral fat loading test were performed in the initiation and also at the end of the study and lipid profile and APO_B_ were measured.

**Results:**

Fasting and postprandial serum triglyceride (TG) decreased significantly with all the three treatment groups with no difference between them in the percent of TG reduction. Although serum total cholesterol decreased significantly in all the three groups of treatment its reduction was more prominent in group C(−38.1% ± 11.2%in group C vs. -16.5% ± 19.6% and −7.2% ± 10.7% in groups B & A respectively, p < 0.0001 ). Fasting serum HDL increased significantly only by Gemfibrozil (23.4% ± 28.4% vs. 6.4% ± 18.9% and 1.8% ± 17.7%, p < 0.05 ). Fasting serum APO_B_ was reduced only in ezetimibe containing groups (B &C).

**Conclusion:**

Adding ezetimibe to gemfibrozil has no additional effect on reducing postprandial TG but ezetimibe can potentiate the effect of low-dose atorvastatin on lowering TG and LDL-c.

## Introduction

Type 2 diabetes is associated with abnormalities in postprandial triglyceride concentrations that are considered as independent cardiovascular risk factors. Based on some studies on humans, both increased secretion and reduced catabolism of apoB containing lipoproteins are responsible for over-accumulation of them in patients with type 2 diabetes and then hypertriglyceridemia [1].

Although fibrates and statins improve many aspects of dyslipidemia in diabetes mellitus, many patients do not touch the goals [2]. So combination therapies with existing lipid-lowering agents are attractive options to control dyslipidemia. Addition of a fibrate to statin therapy can further increase HDL-C and lower TG levels. Between the fibrates, gemfibrozil has the greatest potential to interact with statins. However, this combination is associated with an increased risk of side effects, particularly myopathy and abnormal liver function tests [3, 4].

A new therapy for dyslipidemia treatment is a selective intestinal cholesterol absorption inhibitor, ezetimibe. Ezetimibe inhibits the intestinal absorption of dietary and biliary cholesterol without interfering with the absorption of fat-soluble vitamins. Ezetimibe is a safe and well-tolerated treatment without clinically important drug interactions. Ezetimibe is assumed to produces significant reductions in LDL-C and triglyceride especially when uses in combination with other lipid-lowering agents [5]. It was shown that the co-administration of ezetimibe with other lipid lowering agents can be generally well tolerated. For example, co-administering ezetimibe with on-going simvastatin 10 or 20 mg treatment allowed more hypercholesterolemic patients with chronic heart disease to reach the LDL-C treatment target [6] or a 6-week ezetimibe and simvastatin therapy, compared to simvastatin alone, was presented to have a positive influence on both fasting and postprandial lipoprotein profile in type 2 diabetic patients by favoring the production of cholesterol-poor chylomicrons and VLDL particles that have less atherogenic potential [7].

The potential effects of ezetimibe have been scarcely evaluated and few data are available in type 2 diabetes patients with hypertriglyceridemia. To determine the effect of co-administering ezetimibe with statin or fibrate on fasting and postprandial lipid profile especially postprandial TG concentrations of patients with DM, a double clinical trial has been designed.

## Materials and methods

This study is a randomized; double-blind clinical trial approved by the Ethics Committee of Zanajn University of Medical Sciences, and registered in Iranian registry of clinical trials (http://www.irct.ir) with this code: IRCT201105311179N5.

### Subjects

Seventy-five patients with type 2 DM were selected randomly from the patients who referred to diabetes clinic of vali-e-asr hospital, a referral academic hospital, in Zanjan. All the subjects were less than 60 years old, with acceptable control of DM(HbA1c < 8%) in the last 6 months and fasting TG levels more than 250 mg/dl, LDL-C less than 190 mg/dl and had no medication which affects serum lipid concentrations. Those people with renal dysfunction (GFR < 60 ml/min) or other co-morbidities such as acute or chronic liver disease, thyroid dysfunction, cushing syndrome, acromegaly and malignancy were excluded. After an initial two weeks to test eligibility of the patients 59 subjects approved to enter in the study. Written informed consent was obtained from all the subjects before the study.

An information form including demographic and medical data of the subjects was completed for all of the participants. To analyze the dietary content for the subjects, three samples of 24 –hour food recall forms were used at the beginning and also after the intervention. Calorie intake and macronutrient content of the participants' diet were analyzed by nutritionist IV version 3.5.2 software. Physical activity score of the subjects was determined by a standard short form of previous 7 –day physical activity questionnaire [8] before and after receiving the treatments.

### Measurements

Height, weight and waist circumference of the patients were measured by standard methods and body mass index (BMI) was calculated for all the participants. Blood pressure was measured two times with a 15- minute interval in sitting position and the mean of them was recorded for each of the patients. All the measurements repeated after the end of the study.

Blood samples were collected at baseline to measure serum lipid profile (TG, total cholesterol, LDL-C, HDL-C), fasting plasma glucose (FPG),thyroid stimulating hormone(TSH), liver function tests (AST, ALT, Alkaline phosphatase), serum creatinine concentration(Cr), APO _B_ and HbA1c_._ All blood samples for the assessment of lipid profile were obtained after 14 hours of fast. All the laboratory measurements were conducted at the central laboratory of Vali-e-asr Hospital.

Serum total cholesterol, TG,LDL-C, Cr, AST, ALT, Alp and FPG were measured enzymatically using photometric method by autoanalyser [Minbray (BS-120)] and Pars Azmun kits. HDL-C was measured after precipitation of the apolipoprotein B-containing lipoproteins (LDL-C and very low density lipoprotein [VLDL]) in whole plasma by heparin-manganese chloride. LDL-C was calculated by the method of the Friedewald equation in cases with TG concentrations less than 400 mg/dl. Apolipoprotein (apo) B were determined by Immuno Turbidimetry using ROCH kit and COBAS Integra device. HbA1c was measured in whole blood by borolated affinity method and nyco card kits.

### Oral fat load (cookie test)

For fat loading, a cookie test was performed after overnight fasting for at least 14 h. The cookie consisted of 66 g carbohydrate (60 g wheat and corn contained bread), 41 g fat with 25 g saturated fat and 6.5 g protein(50 g butter + 45 g date palm) for a total of 650 kcal [9]. The participants were instructed to ingest the cookie within 15–20 min. Time measurement was started when half of the cookie had been ingested. Participants remained supine during the test and were only allowed to drink water. Venous blood samples were drawn 4 h after the fat load for serum TG, total cholesterol, LDL, and HDL. Samples were immediately put on ice. Plasma was isolated and stored at -70^C^ for further analyses.

### Study protocol

At the initiation of the study, qualifying patients were randomly assigned to one of three treatment groups: gemfibrozil + placebo, gemfibrozil + ezetimibe and atorvastatin + ezetimibe. Each of the patients received a package including the drugs and a text guide to teach how to use the medications for 6 weeks. The investigator was blind of the content of the packages. Package a contained gemfibrozil capsules [(300 mg) Abidi Laboratory Company] plus placebo tablets apparent like ezetimibe. Atorvastatin tablets [(10 mg) Sobhan Daru Company] plus ezetimibe tablets [(10 mg) Alborz Daru Company] were packed in Package B and package C included gemfibrozil capsules plus ezetimibe tablets.

Group A received gemfibrozil 1200 mg/d plus one tablet of placebo, group B treated with gemfibrozil 1200 mg/d plus one tablet of ezetimibe (10 mg/d) and group C received atorvastatin tablets (10 mg/dl) plus ezetimibe tablets (10 mg/dl) [10]. All the subjects treated for 6 weeks. Because of existence of powerful evidences for the more effectiveness of combination therapy with ezetimibe and statin compared to monotherapy with these medications on lowering LDL-c and TG with lower side effects, we omitted the group taking atorvastatin alone or ezetimibe alone in our study.

Safety was evaluated through patient reports for adverse events, investigator observations, laboratory tests, physical examinations, and vital signs. Investigators were not blinded to safety laboratory results. Possibility of direct contact with the physician was established to answer patients' questions. We considered hepatic side effects for the medications when rises in AST or ALT were more than three times of the upper limit of normal range and reconfirmed by a second evaluation. All the subjects with significant side effects advised to discontinue the medications and the data were recorded.

After 6 weeks of the treatment, patients were recalled again for reevaluation of the variables have been measured before the intervention including anthropometric and clinical measures, oral fat load test and all the laboratory tests except TSH and Cr. complement of 24–hour food recall form and previous seven –day physical activity questionnaire were done again for all the participants.

### Statistical analysis

A sample size of 25 in each group was calculated based on 0.25 differences of TG means and assuming a standard deviation of 0.3, a power of 80%, and a significance level of 5%. The power for 16 patients in each group, using the above information, was 65%.

Data are expressed as mean ± SD. We analyzed mean percentage changes from baseline in fasting and postprandial TG, total cholesterol, LDL-C, HDL-C and Apo B after 6 weeks of treatment between the three groups, using an analysis of variance (ANOVA). Differences between the two groups were evaluated by t-tests. Paired t-test was used to evaluate the changes in one group before and after the intervention. Non-parametric tests were used to analyze the data of variables without normal distribution.

Simple and multivariate regression analysis was used to determine the correlations between the variables. P values less than 0.05 were considered statistically significant. The statistical analysis was performed according to standard methods using the Statistical Package for Social Sciences software 16.

## Results

### Patient accounting and characteristics at baseline

A total of 75 patients were screened at baseline for this study. Of these, 59 patients were eligible and enrolled. Totally twelve patients excluded and did not complete the study including: two with side effects that both of them were gastrointestinal complications and were in group A (gemfibrozil alone), and ten who didn't want to do oral fat load test for final evaluation. The remaining 47 eligible patients were assigned in 3 groups as follows: 14 subjects in group A treated with gemfibrozil + placebo, 16 patients in group B who received ezetimibe + gemfibrozil, and 17 participants in group C with receiving ezetimibe + atovastatin. With this number of participants the power of the trial estimated to be 80%. Patient characteristics at baseline are shown in Table 1.

**Table 1:** **Baseline clinical and laboratory characteristic of participants in the three treatment groups**

Variables	Group A	Group B	Group C	Total	P value
	(Gemfibrozil)	(Ezetimibe + Gemfibrozil)	(Ezetimibe + Atorvastatin)		
	(N = 14)	(N = 16)	(N = 17)	(N = 47)	
**Clinical characteristics**					
Sex					
Male/Female	5/9	5/11	5/12	15/32	0.93
Age (y)	54.2 ± 8.2	51.3 ± 10.5	50.2 ± 11.3	51.8 ± 10	0.54
DM duration (y)	8.1 ± 5.7	5.2 ± 3.3	6.1 ± 5.6	6.4 ± 5	0.28
Hypertension (%)	10 (71.4)	8 (50)	8 (47.1)	26 (55.3)	0.34
BMI (kg/m2)	30 ± 3.8	31.3 ± 4.2	29.8 ± 4.5	30.4 ± 4.1	0.54
Cigarette smoking (%)	1 (7.1)	1 (6.2)	3 (17.6)	5 (10.6)	0.5
Retinopathy (%)	4 (6.28)	2 (5.12)	1 (9.5)	7 (9.14)	0.19
Neuropathy (%)	4 (6.28)	5 (2.31)	7 (2.41)	16 (34)	0.73
Cardiovascular disease (%)	1 (6.2)	1 (6.2)	0	2 (4.3)	0.54
**Laboratory test**					
FPG (mg/dl)	110 ± 25	130 ± 37	145 ± 36		.019^1^
FTG	343 ± 128	351 ± 121	307 ± 117		0.56
F Chol	231 ± 47	207 ± 36	220 ± 36		0.3
F HDL-c	40 ± 8	35 ± 8	45 ± 13		0.048^1^
HbA1c	7.7 ± 1.9	7.8 ± 1.2	7.9 ± 0.9		0.88
Apo A	147 ± 19	144 ± 20	156 ± 26		0.33
Apo B	100 ± 19	93 ± 19	92 ± 16		0.36
PL TG	451 ± 155	462 ± 138	429 ± 141		0.81
PL Chol	228 ± 44	209 ± 36	222 ± 36		0.44
PL HDL-c	36 ± 8	33 ± 7	42v15		0.85

Abbreviations: *DM* Diabetes Mellitus, *BMI* Body mass Index, *FPG* Fasting Plasma Glucose, *FTG* Fasting Triglyceride, *F Chol* Fa Post Fat Load sting Cholesterol, *F HDL-c* Fasting High Density Lipoprotein, *PL TG* Post Fat Load Triglyceride, *PL Chol* Post Fat Load Cholesterol, *PL HDL-c* Post Fat Load High Density Lipoprotein.

Mean age of all subjects was 51.8 ± 10.1 years old with mean duration of DM 6.4 ± 5 years. Thirty two of the participants were taking oral hypoglycemic agents for their diabetes control, one subject was controlled with insulin and twelve were receiving combination therapy with insulin and oral hypoglycemic agents. Two of the patients were controlled with diet alone. There was no considerable difference between the three groups regarding the mode of treatment to control hyperglycemia.

No significant differences were found between the three intervention groups for basal serum levels of fasting and postprandial (after fat load test) lipid profile, HbA1c, TSH, Cr and apoB. Data about the diet content and physical activity score of the participants is shown in Table 2. There were no significant changes in calorie intake and physical activity of the subjects within and between the groups before and after the intervention except for group c that had higher calorie intake from fat after the intervention.

**Table 2:** **Diet content and physical activity score of subjects within and between the groups before and after the intervention**

Variables	Group A			Group B			Group C			P value (between groups)
	(Gemfibrozil)			(Ezetimibe + Gemfibrozil)			(Ezetimibe + Atorvastatin)			
	(N = 14)			(N = 16)			(N = 17)			
	Before	After	P-value	Before	After	P- value	Before	After	P- value	
**Total calorie intake (kcal)**	1330 ± 607	1333 ± 527	0.968	1390 ± 435	1435 ± 539	0.69	1281 ± 454	1369 ± 448	0.394	0.82
**Percent of calorie intake from fatty acid (%)**	21.4 ± 7.5	17.5 ± 6.6	0.13	14.7 ± 6.6	17.7 ± 8.2	0.15	15.7 ± 5.4	20.5 ± 9.6	0.05	0.018^1^
**Fat intake(g)**	31.6 ± 16.4	26.7 ± 13.7	0.32	23.8 ± 13.5	29.9 ± 19	0.2	23.6 ± 15.2	30.1 ± 15.8	0.1	0.26
**Carbohydrate intake (g)**	202.9 ± 95	234.6 ± 91	0.12	232.7 ± 74	243.1 ± 93	0.65	221.4 ± 74	213.3 ± 89	0.14	0.63
**Protein intake (g)**	59.7 ± 31.3	52 ± 30.2	0.31	63.5 ± 36.8	52.4 ± 19.8	0.15	49.5 ± 19.4	62.4 ± 32.4	0.14	0.38
**Physical activity score**	−2.6 ± 13.6	0.9 ± 17.9	0.62	−2 ± 10.5	−4.1 ± 8	0.48	7.5 ± 18.2	5.1 ± 18.5	0.64	

1-P value <0.05 is considered significant.

Administration of ezetimibe, atorvastatin and gemfibrozil was well tolerated.

In general, the types of adverse events resulting in treatment discontinuation or interruption were no more common or severe in any treatment group. No patient died during the study. No significant elevations in hepatic enzymes were seen, and no cases of hepatitis, jaundice, or other clinical signs of liver dysfunction were reported.

### Fasting lipid profile after treatment

Six weeks treatment of hyperlipidemia in 47 DM patients resulted in a significant decrease in serum fasting TG level in all of the three groups (P < 0.0002). In compare to group C the reduction rate for fasting TG was more prominent in groups A and B which containing gemfibrozil. Moreover no additional effect was seen in group B containing ezetimibe in comparison with group A regarding to reducing fasting serum TG(−175 ± 153 mg/dl reduction in serum fasting TG by ezetimibe plus gemfibrozil, and −176 ± 80 mg/dl by gemfibrozil alone).

Total serum cholesterol also decreased in all of the groups (P < 0.05), but was more remarkable in those received ezetimibe co-administering atorvastatin (group C) (38 ± 11% reduction in group C versus 7% reduction in group A and 16% reduction in group B, P:0.0001). LDL-c decreased significantly in group B and C who were treated with ezetimibe plus gemfibrozil or atorvastatin (p < 0.0001), but a non significant increment in LDL-c was seen with gemfibrozil alone (group A). Apo B reduction was seen in groups B and C which containing ezetimibe (p: 0.04) and was more prominent in group C (Table 3). HDL-c increased significantly only in group A who were under treatment with gemfibrozil alone (p: 0.014).

**Table 3:** **Comparison of fasting and post fat load laboratory variables before and after treatment in each of the three treatment groups**

Variable (fasting)	Group A			Group B			Group C		
	(Gemfibrozil)			(Ezetimibe + Gemfibrozil)			(Ezetimibe + Atorvastatin)		
	(N = 14)			(N = 16)			(N = 17)		
	Before	After	P value	Before	After	P value	Before	After	P value
TG (mg.dl)	351 ± 121	175 ± 85	<0.0001^1^	343 ± 128	168 ± 53	<0.0001^1^	307 ± 117	202 ± 85	0.002^1^
T-Chol (mg.dl)	207 ± 36	191 ± 31	0.034^1^	231 ± 47	188 ± 41	0.007^1^	220 ± 36	136 ± 32	<0.0001^1^
HDL (mg.dl)	35 ± 8	42 ± 5	0.014^1^	40 ± 8	42 ± 7	0.35	45 ± 13	45 ± 13	0.98
LDL (mg.dl)	101 ± 48	113 ± 35	0.15	125 ± 41	112 ± 39	0.38	116 ± 29	50 ± 17	<0.0001^1^
FBS (mg.dl)	130 ± 32	131 ± 35	0.86	110 ± 1.9	118 ± 0.8	0.32	145 ± 36	138 ± 48	0.58
HbA_1C_ (%)	7.8 ± 1.2	7.9 ± 1.4	0.65	7.7 ± 1.9	7.5 ± 0.8	0.63	7.1 ± 0.9	7.9 ± 1.6	0.81
APO_A1_ (mg.dl)	144 ± 20	138 ± 22	0.23	147 ± 19	136 ± 35	0.019^1^	156 ± 26	151 ± 27	0.01
APO_B_ (mg.dl)	93 ± 19	94v20	0.6	100 ± 19	91 ± 22	0.04	92 ± 16	55 ± 13	<0.0001^1^
AST (IU.L)	26 ± 9	30 ± 8	0.076	29 ± 11	29 ± 13	098	22 ± 8	23 ± 5	0.56
ALT (IU.L)	25 ± 12	25 ± 13	0.9	26 ± 12	24 ± 13	0.73	21 ± 10	23 ± 15	0.68
Alk.p (IU.L)	274 ± 135	234 ± 105	0.04^1^	207 ± 63	183 ± 63	0.1	204 ± 66	204 ± 50	0.97
**(post fat load)**									
TG (mg.dl)	436 ± 138	221 ± 109	<0.0001^1^	451 ± 155	216 ± 66	<0.0001^1^	429 ± 141	267 ± 104	<0.0001^1^
Choesterol (mg.dl)	209 ± 36	188 ± 33	<0.0001^1^	228 ± 44	190 ± 40	<0.0001^1^	222 ± 36	137 ± 33	<0.0001^1^
LDL-c (mg.dl)	101 ± 39	106 ± 33	0.59	109 ± 38	106 ± 37	0.79	106 ± 32	47 ± 18	<0.0001^1^
HDL-c (mg.dl)	33 ± 7	40 ± 5	0.009^1^	36 ± 8	40 ± 8	0.033^1^	42 ± 15	43 ± 13	0.776

1-P value <0.05 is considered significant.

2-*TG* triglyceride, *T_Chol* total cholesterol, *HDL* High Density lipoprotein, *LDL* Low Density lipoprotein, *FPG* Fasting Plasma Glucose, *HbA*_*1*_*C* Hemoglubin A_1_C, *TSH* Thyroid Stimulating Hormone, *APO*_*A1*_ Apoprotein _A1_, *APO*_*B1*_ Apoprotein _B1_.

### Postprandial lipid profile after treatment

Post fat load test TG level reduced significantly in all the three intervention groups (Table 3). There was no difference between the groups for the reduction rate of postprandial TG level (52 ± 17% reduction in group A versus 46 ± 24% in group B and 35 ± 24% in group C,P:0.1).

Post fat load test total cholesterol and LDL-c levels showed significant reduction in all the three groups of intervention, but was more greater by ezetimibe plus atorvastatin (38 ± 11% reduction in postprandial total cholesterol in group C versus 15 ± 17% reduction in group B and only 9 ± 11% in group A, p: 0.0001) (Table 3). Although HDL-c after fat load test increased in all the three groups specially in group A the difference between the groups was not statistically significant (Table 3).

Finally, Multivariate regression analysis demonstrated that the reduction rate of fasting and postprandial TG level was inversely correlated with baseline serum fasting and postprandial TG concentration (p: 0.008). Other confounding factors like age, sex, duration of diabetes, BMI, baseline calorie intake and percent of calorie intake from fat had no significant effect on the changes of serum TG with the treatments (Table 4).

**Table 4:** **Result of multivariate regression analysis for percent of reduction in fasting and post fat load triglyceride concentration with the treatments**

Independent variable	Dependant variable	Multivariate regression	
		Beta	P-value
**Percent of fasting TG reduction**	Gender	0.195	0.165
	Age	−0.216	0.141
	Baseline BMI	0.247	0.067
	Baseline calorie intake	0.155	0.31
	Baseline calorie intake from fatty acid	0.031	0.807
	Baseline fasting TG	−0.368	**0**.**008**^**1**^
**Percent of postprandial TG reduction**	Gender	0.226	0.127
	Age	0.136	0.38
	Baseline BMI	0.26	0.07
	Baseline calorie intake	0.109	0.491
	Baseline calorie intake from fatty acid	0.043	0.748
	Baseline fasting TG	−0.314	**0**.**035**^**1**^

1-P value <0.05 is considered significant.

## Discussion

A gap between lipid goals and what we reach in clinical practice leads us to investigate and find the best combination protocol for diabetic patients with hyperlipidemia. In this trial, fasting and postprandial serum triglyceride (TG) and cholesterol decreased significantly with all of the three modes of treatment. Adding ezetimibe to gemfibrozil not only had no further effect on lowering fasting TG but also might attenuate the beneficial effect of gemfibrozil on postprandial TG and HDL-C. However co-administering gemfirozil and ezetimibe improved the effect of gemfibrozil on total cholesterol, LDL-c and Apo-B concentrations. The best effect on serum fasting and postprandial total cholesterol and also LDL-c was seen with combination of ezetimibe and atorvastatin (38% reduction rate).

Efficacy, safety and tolerably are the ideal characteristics of a second drug to complement either statins or fibrates as the first-line treatment options for dyslipidemia in diabetic patients [7, 11].

Ezetimibe is a hypocholesterolemic drug that selectively inhibits intestinal cholesterol absorption. Beside the effect on LDL-c levels, an additional property related to reducing metabolism of long-chain fatty acids and blocking apo-B48 formation might be the mechanism of chylomicron lowering effect of ezetimibe [12]. Furthermore, ezetimibe might reduce cholesterol availability for VLDL endogenous synthesis in the liver and affect hypertriglyceridemia [13]. Also, new animal based researches revealed that treatment with ezetimibe not only inhibits cholesterol uptake, but may also modulates intestinal function and improves dietary lipids handling and reduces chylomicron production. These, in turn, promote decreases in fasting and postprandial lipid levels and improvements in glucose homeostasis [14].

Studies on ezetimibe monotherapy in randomized prospective trials have been shown that ezetimibe decreases fasting and postprandial TG and apoB levels after the fat loading test [15, 16].

Statins are considered to play a main role in lowering LDL-C. High dose of statins are associated with more side effects like myopathy and rising liver enzymes. Furthermore, most of diabetic patients suffer from combined hyperlipidemia. Therefore combination therapy with lipid-lowering drugs is mandatory in many of diabetic patients. Although no differences were found in fasting lipid profiles between low-dose simvastatin with ezetimibe and monotherapy with high-dose of simvastatin in one study [17] when co-administered with atorvastatin, ezetimibe resulted significant incremental reductions in LDL-C and triglycerides and increased in HDL-C and was well tolerated, with a safety profile similar to atorvastatin alone or to placebo [18, 19].

In many other studies with different designs and populations of patients, ezetimibe co-administered with all doses of current statins caused significant incremental reductions in LDL-C and allowed more patients to reach LDL-C goal compared to the respective dose of statin monotherapy. For example in Japanese patients with type 2 diabetes or impaired glucose tolerance and coronary artery disease, adding ezetimibe (10 mg/day) to atorvastatin (10 mg/day) significantly improved the lipid profile compared with atorvastatin monotherapy at 20 mg/day [20, 21]. We also found a significant reduction in the content of total cholesterol, LDL and TG after combination therapy with atorvastatin and ezetimibe both at fasting and postprandial phase, although the percent of calorie intake from fat was higher after the intervention in the participants.This significant reduction of TG and LDL in the presence of higher fat intake in group C shows a potent lipid- lowering effect of combination of ezetimibe and statin. One of the limitations of the present study was absence of a group with statin mono therapy. Because of existence of powerful evidences for the more effectiveness of combination therapy with ezetimibe compared to statin monotherapy on lowering LDL-c and TG with lower side effects, we omitted the group taking atorvastatin alone in our study.

Consistent with previous findings [16, 17, 22, 23], our interesting finding showed statistically significant postprandial TG reduction response rather than fasting TG reduction in all types of the interventions. Routinely, we measured triglyceride levels in fasting blood specimens; but evidence shows that postprandial triglycerides further increase oxidative stress and is considered as a contributing factor to endothelial dysfunction, atherosclerosis, and cardiovascular disease.

The mechanisms of fasting and postprandial increases of TG and intestine-derived lipoproteins in diabetes are supposed to be slower clearance of these particles and/or a reduced activity of lipoprotein lipase (LPL) specifically related to type 2 diabetes [24, 25].

When triglycerides are high, the first approach can be intensified using an LDL lowering and reaching the low-density lipoprotein cholesterol (LDL–C) goal of <130 mg/dl. The second approach used with caution is the addition of nicotinic acid or a fibrate drug [26].

Fibrate is a PPAR-α agonist and used to treat hypertriglyceridemia and mixed dyslipidemia. It is approved for the treatment of dyslipidemia commonly in patients at high risk of cardiovascular disease, including Type 2 diabetes and/or metabolic syndrome. Fibrates lowers triglycerides, raises HDL-cholesterol and decreases concentrations of small LDL-cholesterol particles and apolipoprotein B. Fenofibrate is particularly successful for reducing postprandial VLDL and LDL particle and oxidative stress and inflammatory response after a fatty meal [27]. Fibrate therapy results in significant reductions in fasting triglyceride concentrations and has been shown to reduce fibrinogen and C-reactive protein in inflammatory mediators [28].

With the best of our knowledge, this is the first study on the effectiveness of combination therapy with a fibrate (Gemfibrozil) and ezetimibe on reducing fasting and postprandial TG levels. Most of the previous studies have focused on the effectiveness of combination therapy with ezetimibe and statines. The others have investigated the effect of fibrate monotherapy on postprandial TG concentration or the effect of coadminestering ezetimibe and fenofibrate on fasting and not postprandial TG [26, 29]. We found that the reduction rate of TG after treatment is correlated negatively to the baseline TG levels. Such result has been previously reported by Farnier in a review article [29].

We acknowledge some limitations of our study. Due to the short duration of the present study (6 weeks), conclusions about long-term efficacy and safety cannot be made. Additionally, patients were recruited with specific inclusion and exclusion criteria and thus extension of these results to other populations should be done with caution. However, long-term efficacy, durability and tolerability have been demonstrated in studies up to 48 weeks with ezetimibe co-administered with either simvastatin [30] or atorvastatin [31].

## Conclusion

Although adding ezetimibe to gemfibrozil has no additional effect on reducing postprandial TG but ezetimibe can potentiate the effect of low-dose atorvastatin on lowering TG and LDL-c. Based on the results of this study, a fibrate agent (gemfibrozil) is one of the best to control postprandial hypertriglyceridemia. For patients with diabetes mellitus type 2 and pure hypertriglyceridemia, monotherapy with gemfibrozil might be a good choice especially in those with low HDL-c.

## Abbreviations

TG: Triglyceride
LDL: Low density lipoprotein
HDL: High density lipoprotein
VLDL: Very low density lipoprotein
FPG: Fasting plasma glucose
ALT: Alanine aminotransferease test
AST: Asparate aminotransferase test
Alk P: Alkaline phosphatase
HbA1C: Haemoglubin A1_C_
Cr: Creatinine
CRP: C-reactive protein
DM: Diabetes mellitus.
